# Late anastomotic perforation following surgery for gastric neuroendocrine tumor complicated by perforated duodenal ulcer: a case report

**DOI:** 10.7555/JBR.27.20110109

**Published:** 2012-03-29

**Authors:** Jun Han, Zhenyu He

**Affiliations:** Department of General Surgery, the Second Affiliated Hospital of Nanjing Medical University, Nanjing, Jiangsu 210011, China

**Keywords:** anastomotic perforation, neuroendocrine tumor, complications

## Abstract

Neuroendocrine tumors (NETs) are a group of neoplasms that are characterized by the secretion of a variety of hormones and diverse clinical syndromes. NETs are considered to be rare, but the incidence of NETs has increased rapidly in recent years. NETs provide a clinical challenge for physicians because they comprise a heterogeneous group of malignancies with a wide range of morphological, functional, and behavioral characteristics. Subtotal gastrectomy with Billroth II reconstruction is the mainstay of therapy in the management of gastric NETs complicated by perforated duodenal ulcer. Late perforation of anastomotic stoma as a long-term complication has been rarely reported. Here, we report a case of anastomotic perforation 5 years after subtotal gastrectomy due to perforated duodenal ulcer and gastric NETs.

## INTRODUCTION

Neuroendocrine tumors (NETs) are considered to be rare, heterogeneous and complex neoplasms[1]. They are characterized by their abilities to produce bioactive substances which can induce diverse clinical syndromes[2]. According to a recent study by the Tuscan Cancer Registry, the overall incidence of NETs in 2005 was 1.6 and 2.1 per 100,000 persons per y among men and women, respectively. The anatomic distribution of NETs includes the lung (25.7%), small intestine (23.5%), appendix (10.9%), colon (10.3%), pancreas (9.4%), stomach (7.4%) and rectum (5.2%)[3]. However, an overview of the recent developments in NETs of the gastroenteropancreatic tract showed a significant increase in the incidence of these tumors[4].

Subtotal gastrectomy with Billroth II reconstruction is the mainstay of therapy in the management of gastric NETs complicated with perforated duodenal ulcer. Common complications related to subtotal gastrectomy are postoperative bleeding, anastomotic leakage, pancreatic juice leakage, intra-abdominal abscess, intestinal obstruction, and wound dehiscence. Late perforation at the anastomotic site as a long-term complication of subtotal gastrectomy has been rarely reported, especially following surgery for NETs. Here, we report a case of anastomotic perforation after subtotal gastrectomy with Billroth II reconstruction owing to gastric NETs complicated with perforated duodenal ulcer 5 years ago.

## CASE REPORT

**Fig. 1:**
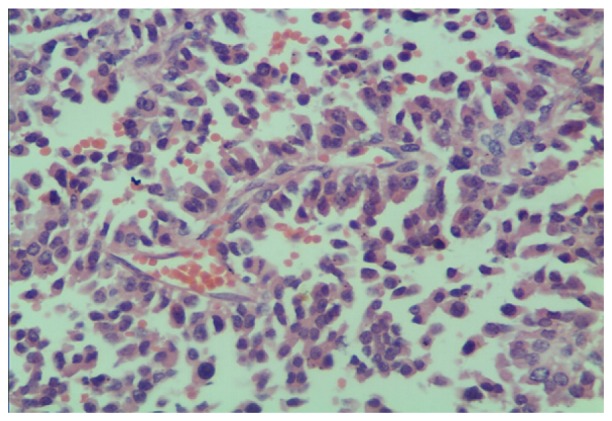
H&E staining of the lesser curvature of the stomach (H&E, 400×). The image shows well-differentiated gastric neuroendocrine tumor cells around blood vessels with abundant red blood cells in a 41-year old male patient with late anastomotic perforation following surgery for gastric neuroendocrine tumor.

A 41-year-old male patient, with progressive abdominal pain during the previous 48 hours, was admitted to the Department of General Surgery, the Second Affiliated Hospital of Nanjing Medical University. No fever, nausea, vomiting, melena and jaundice were observed during the period. He had a history of subtotal gastrectomy with Billroth II reconstruction for perforation at the site of the duodenum and a mass near the lesser curvature of the stomach at the other hospital. Pathological and immunohistochemical examinations of the excised specimen (***Fig. 1***) revealed that the mass near the lesser curvature of the stomach was poorly differentiated gastric NETs (type III). Serum gastrin content was normal and Zollinger-Ellison syndrome was excluded. The perforation was due to duodenal ulcer rather than a malignancy. The patient was examined regularly with abdominal CT for 2 years after the operation, but no metastasis was detected during the period. No further follow-up was performed during the subsequent 2 years. The patient visited our hospital for sudden abdominal pain and vomited one years ago. CT scan revealed multiple liver metastases located at both the left and right lobes of the liver with coeliac lymph node enlargement. Meanwhile, serum gastrin content was still normal. Owing to the widespread metastasis, liver metastases were treated through trans-arterial chemoembolization (TACE) with 5-Fu 0.5 g, epirubicin 20 mg, and cisplatin 40 mg combined with lipiodol four times rather than surgery. Two courses of intravenous chemotherapy at the Department of Oncology with irinotecan 100 mg and cisplatin 40 mg were given to him owing to elevated serum neuron-specific enolase (NSE) levels. He was discharged from the Department of Oncology when the level of serum NSE returned to normal, which was also allowed by his general state of health.

**Fig. 2:**
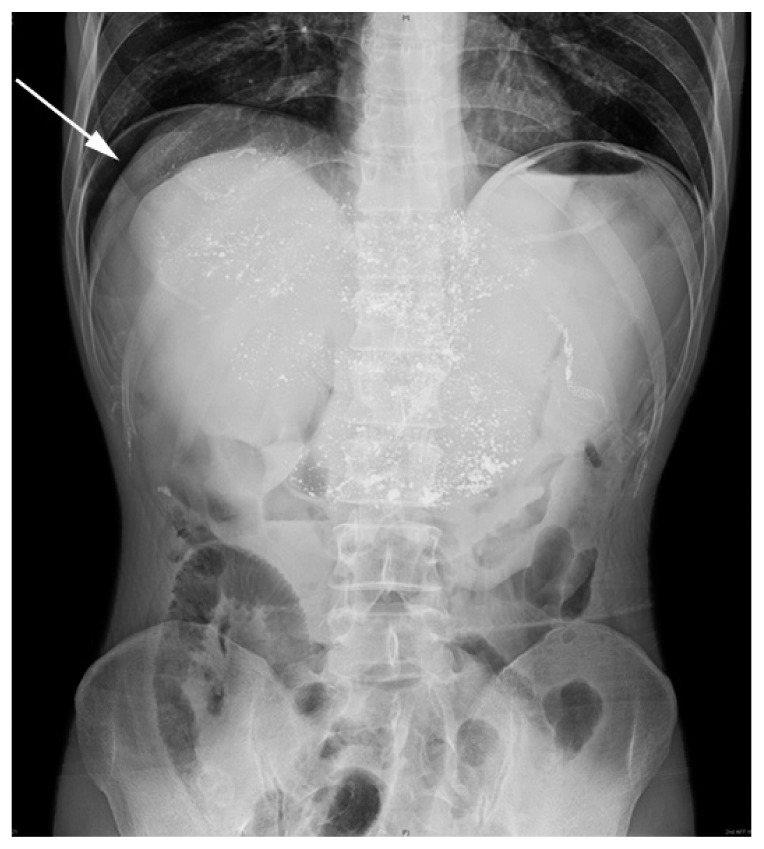
Plain X-ray of the abdomen revealed little free gas in the subphrenic spaces (arrow) in a 41-year old male patient with late anastomotic perforation following surgery for gastric neuroendocrine tumor.

**Fig. 3:**
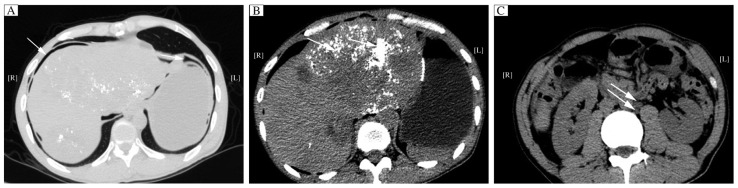
Abdominal pre-contrast CT Scans (PCT). PCT shows little free gas in the subphrenic spaces and around the gastric remnant (A, arrow), multiple liver metastases with inhomogeneous iodized oil drop (B, arrow) and multiple enlarged lymph nodes near the abdominal aorta (C, arrow).

On his arrival at the hospital, he was previously misdiagnosed as intestinal obstruction or acute pancreatitis considering that his vital signs were normal except for a poor mental status. A gastrointestinal decompression tube was immediately filled with about 400 mL black gastric juices that drained out of the stomach. Plain X-ray of the abdomen revealed little free gas in the subphrenic spaces (***Fig. 2***). Abdominal CT scan showed some free gas in the subphrenic spaces, inhomogeneous iodized oil on the liver and multiple enlarged lymph nodes near the abdominal aorta (***Fig. 3***). We scheduled laparoscopic exploration in view of the perforation. Hepatomegaly and edema of the small intestines combined with extensive adhesions in the peritoneal cavity were observed. After careful separation of the adhesions, laparoscopic exploration revealed a perforation of 1.5 cm in diameter at the site of previous gastroenterostomy. Gastric juice along with food debris could be seen coming out from the site of perforation when we were conducting the operation. Based on these findings, we decided to close the perforation site directly through the open operation rather than laparoscopic exploration because of severe intra-abdominal adhesions. We successfully repaired the perforation and a drainage tube was placed. The patient had a relatively uneventful recovery except slight intestinal tympany. He was discharged from the hospital 15 day after surgery based on his condition and he remained alive after two months of follow-up.

## DISCUSSION

Gastric NETs were classified by the World Health Organization (WHO) into the following three types. Type I tumors are well-differentiated NETs. These tumors occur in coincidence with chronic atrophic gastritis, as single or multiple small tumors. The prognosis of type I tumors is excellent with no metastasis. Type II tumors are well-differentiated neuroendocrine carcinomas. These tumors are part of the MEN-1 syndrome and associated with Zollinger-Ellison syndrome. The prognoses for most of these patients are good. Type III tumors are poorly differentiated neuroendocrine carcinomas. These tumors are sporadic and may develop metastasis, with an overall poor prognosis[5]. Well-differentiated gastric NETs are regarded as gastric carcinoids, which account for only 8.7% of all gastrointestinal carcinoids[6]. Although NETs are considered to be rare, the incidence of NETs has risen five-fold during the last three decades in the US while a similar increase was reported by the Norwegian Registry of Cancer (NRC)[7],[8]. Turaga et al.[9] believed that the remarkable increase of the incidence was related to an increase in awareness and advances in pathological diagnosis, classification, tumor imaging with endoscopic ultrasound and somatostatin receptor fusion imaging.

For optimal management of NETs, the type, biology and stage of the tumor as well as the individual situation of the patient should be considered[10]. As for the treatment of NETs, poorly differentiated NETs with a poor prognosis are mainly treated with chemotherapy using different chemotherapeutic agents[11]. Patients with well-differentiated NETs are predominantly treated by surgical excision, which is the only potentially curative treatment for well-differentiated NETs[12],[13]. In recent years, a multidisciplinary approach involving surgical oncology, medical oncology, endocrinology, diagnostic radiology, interventional radiology as well as experienced nurses has been advocated for diagnosis and therapy of NETs[14].

The present case we report here was identified to be poorly differentiated gastric NETs complicated with perforated duodenum by pathological and immunohistochemical examinations 5 years ago. Subtotal gastrectomy with Billroth II reconstruction was performed and no early complications occurred.

Common complications related to subtotal gastrectomy with Billroth II reconstruction are postoperative bleeding, pancreatitis, intestinal occlusion, anastomotic leakage, pancreatic juice leakage, intra-abdominal abscess, intestinal obstruction, wound infection, and wound dehiscence[15]. Early perforations of anastomoticstoma are mostly reported in patients with anastomotic ulcers[16]. Late perforation at the site of the anastomotic stoma is rarely reported. To our knowledge, no anastomotic perforation has been reported before as a late complication after subtotal gastrectomy with Billroth II reconstruction due to gastric NETs complicated with perforated duodenal ulcer.

In our opinion, the possible reasons for anastomotic perforation in this case are as follows: Anastomotic stoma was fully infiltrated by the metastatic tumor. A newly gastric ulcer emerged at the site of the anastomotic stoma. The anastomotic stoma was edematous and friable after chemotherapy and could rupture after a full meal in any situation mentioned above. Unfortunately, we had no idea of the exact cause because the condition of the patient was so poor that a biopsy could not be performed at the time. This case was misunderstood at the Division of Gastroenterology at first. In order to avoid the same mistakes, rare perforation at the site of anastomotic stoma should be considered when patients complain of acute abdominal pain with board-like rigidity of the abdomen after gastroenterostomy.

In conclusion, anastomotic perforation as a late complication after subtotal gastrectomy for gastric NETs complicated by perforated duodenal ulcer has not previously been reported. We analyzed the possible causes of anastomotic perforation as well as misdiagnosis in this case and pointed out that more information should be collected to develop our knowledge about diagnosis and treatment of this problem.
